# Theoretical and Experimental Study of Joint Osmotic and Electroosmotic Water Transfer through a Cation-Exchange Membrane

**DOI:** 10.3390/ijms232112778

**Published:** 2022-10-24

**Authors:** Anatoly N. Filippov, Svetlana A. Shkirskaya

**Affiliations:** 1 Department of Higher Mathematics, National University of Oil and Gas “Gubkin University”, 119991 Moscow, Russia; 2 Department of Physical Chemistry, Kuban State University, 350040 Krasnodar, Russia

**Keywords:** osmotic and electroosmotic permeability, ion-exchange membrane, cell model, Onsager approach

## Abstract

Using the previously developed cell model of a charged membrane and the principles of linear thermodynamics of irreversible processes (the Onsager approach), exact and approximate (in the case of an ideally selective membrane) analytical formulae for calculating the osmotic and electroosmotic permeability of the membrane in aqueous solutions of 1:1 electrolyte at constant electric current density and concentration gradient were suggested. The formulae have been successfully verified by our own experimental data for the extrusion cation-exchange membrane MF−4SC p.29 in NaCl solution up to concentrations of 3 M. The contribution of electroosmotic and osmotic water fluxes to the total water transport through the mentioned individual perfluorinated ion-exchange membrane under conditions close to the process of electrodialysis concentrating was experimentally estimated. The cases of co- and counter-directed osmotic and electroosmotic water fluxes are studied. A good correspondence between theoretical and experimental results was obtained, which made it possible to determine the physicochemical parameters of the electromembrane system (the diffusion coefficients of individual ions and the coefficient of equilibrium distribution of electrolyte molecules in the membrane matrix, the characteristic exchange capacity of the cell model). The achieved results make it possible to fully characterize existing and promising types of ion-exchange membranes based on the developed cell model of a charged membrane.

## 1. Introduction

Determination of the coefficients of osmotic and electroosmotic permeability of membranes whose matrix carries a fixed charge is an important task in the characterization of both commercially available and new composite and hybrid ion-exchange materials obtained by modifying their properties by adding various organic or inorganic dopants. However, when studying the transport properties of membranes, the question often arises—were experimental measurements of these characteristics carried out correctly? This question can be answered from the point of view of thermodynamics of nonequilibrium processes [1] by applying the well-known Onsager approach, which linearly connects thermodynamic forces (the independent process parameters) and fluxes caused by these forces (the dependent thermodynamic parameters).

As early as 1956, attempts were made to separate the total flux of water carried through the membrane when an external electric field was applied to water in the composition of solvate ion shells and free water [2]. Perfluorinated ion-exchange membranes are approaching in their properties an ideally selective membrane, which has two charged components, and only one of them, namely the counterion, is mobile. The unidirectional flow of counterions generates the unidirectional flow of solvent, i.e., electroosmosis. In [2], the hydration number of the sodium ion in the membrane was found, which is equal to 9 mol H_2_O/*F*_0_ (*F*_0_ is the Faraday constant). The overestimated value obtained was explained by the authors so that it is not the primary solvation number but determines all the water that migrates with the ion when an external electric field is applied—that is, the dynamic hydration number. In [2], it was concluded that direct measurement of electroosmosis in charged membranes is impossible since the total volume of the solution transported by one Faraday of electricity consists of the transfer of “free” water and approximately the same volume of transported ions together with the volume of their solvate shells.

Interest in the research of electroosmotic and osmotic water transfer in ion-exchange membranes is associated with their application in electrodialysis apparatuses, where the fluxes of free and bound water are a negative factor that leads to the dilution of the process solution. It was shown in [3,4] that during the electrodialysis concentrating of electrolyte solutions, the main contribution to the total process of water transfer through a membrane pair is created by electroosmotic water transfer. According to the authors of [3,4], if the contribution of diffusion is 5% and osmosis is 15%, then the contribution of electroosmosis reaches 80%. It was shown in [3,4] that the effect of the diffusion component on the process of electrodialysis concentrating is negligible compared to osmotic and electroosmotic contributions, which limit the maximum concentration of the resulting electrolyte. Therefore, determining the contribution of osmotic and electroosmotic fluxes to the total flux of transported water is an extremely important task. The role of the electroosmotic transport will increase even more when using membranes of various types with high moisture content, as well as during the transition from aqueous solvents to aqueous organic media. In [5], the osmotic water transfer coefficient was calculated for the CMS and ACS membrane pair (Neosepta, Japan), *D*_w_ = 2.04 × 10^−10^ m^2^/s, which is associated with the difference in osmotic pressure caused by the ionic concentration gradient on both sides of the membrane. This value is about an order lower than the diffusion coefficient of water in a pure solution. In the same work, it was shown that the electroosmotic transfer of water is proportional to the applied current density, does not depend on the concentration gradient across the membrane and is 6.4 (±1.5) mol H_2_O/*F*_0_. In [6], the hydration numbers of individual ions and electrolyte solutions were calculated from experimental data of ion and water fluxes obtained in an electrodialysis cell during the concentration of pure and mixed electrolyte solutions. It is shown that the calculated hydration numbers for monovalent ions are slightly overestimated since not only the water of the first hydrate shell is determined from the mentioned experiments. For divalent ions, the hydration numbers differ even more from the literature data. In [7], the total water flux and osmotic permeability for various membrane pairs were also measured from experiments in an electrodialysis cell. The authors of [8] determined the coefficient of self-diffusion of water in proton-exchange membranes based on cross-linked polytetrafluoroethylene (PTFE) films by the method of water permeability labeled with tritium. It was found that with an increase in the crosslinking density of the PTFE matrix, the water content, defined as the number of water molecules per fixed group of sulfonic acid, decreased, and the value of water mobility became smaller.

In a recent paper [9], we once again demonstrated the importance of experimentally measuring the electroosmotic permeability of individual ion-exchange membranes in solutions of various electrolytes using two alternative methods: the volumetric and the gravimetric ones. Their correspondence is shown, and formulae for calculating water transport numbers using a two-phase membrane model are given.

In recent years, alternative energy sources, including solid polymer fuel cells, the heart of which is a perfluorinated cation-exchange membrane of the Nafion type, have become an important subject of research [10,11,12]. Controlling the transfer of water with a proton is a crucial problem in a solid polymer fuel cell with a proton-exchange membrane since water is not only formed as a result of the cathodic oxygen reduction reaction but is also transported by electroosmotic transfer [13,14,15,16]. These phenomena can lead to an uneven state of hydration in the membrane, which can cause the deterioration of the fuel cells. In other words, the perfluorinated membrane tends to dehydrate from the anode side, which leads to a loss of proton conductivity, while flooding from the cathode side can make it difficult to supply the reagent gas. Thus, the regulation of water transport in a proton-conducting perfluorinated membrane is a serious problem in the development of stable operation of fuel cells [17,18,19], and the study of electroosmotic and osmotic transport through Nafion-type membranes is an urgent task for both experimenters and theorists [20,21]

Due to the complexity of experiments to determine the total flux of water, as well as the separate determination of electroosmotic and osmotic fluxes through ion-exchange membranes, the question of the theoretical determination of these characteristics, knowing other properties of membranes, has always been on the agenda [22,23,24,25,26,27,28,29,30,31,32]. However, the task of a theoretical description of the electric transport of water in charged membranes in a wide range of concentrations is also quite complex and requires various assumptions.

Due to the fact that a theoretical assessment of the mechanism of water transfer in membranes is possible only for a membrane pair in an electrodialysis device, the question of determining the contributions of osmotic and electroosmotic water transport to the total water transport through an individual membrane has remained open until recently. To solve this problem, we performed experiments under conditions close to the real electrodialysis process but with an individual membrane of Nafion type (MF−4SC). In this paper, for the first time, we suggested analytical formulae for determining the osmotic and electroosmotic permeability of a real and ideal cation-exchange membrane as a function of an equilibrium concentration of electrolyte. These formulae were obtained based on the cell model of a charged membrane [30,31,32,33,34,35] and methods of nonequilibrium thermodynamics in a linear approximation and have been successfully verified on our own experimental data. The achieved results make it possible to entirely characterize existing and promising types of ion exchange and other types of membranes based on the developed cell model of a charged membrane.

## 2. Results 

**Figure 1:**
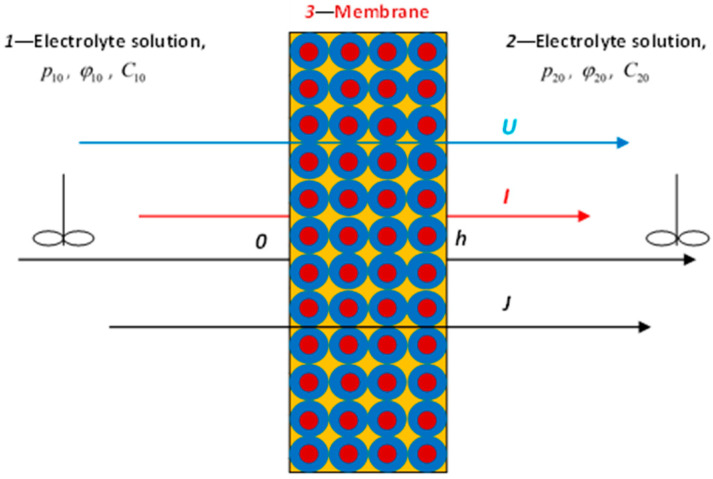
Diagram of the electrolyte solution transfer through a charged membrane.

When modeling nonequilibrium isothermal processes occurring in an idealized membrane consisting of identical porous cells (Figure 1), it is convenient to use gradients of pressure $\nabla p\approx \left(p_{20}-p_{10}\right)/h$, electrical potential $\nabla \varphi \approx \left(\varphi_{20}-\varphi_{10}\right)/h$, and chemical potential (concentration) $\nabla \mu \left(C\right)\approx RT\;\nabla C/C_{0}\approx RT\left(C_{20}-C_{10}\right)/\left(C_{0}h\right)$ as thermodynamic forces. Here *C*_0_ is the equilibrium concentration of electrolyte in the cell, *R* is the universal gas constant, *T* is the absolute temperature, and the indices “1” and “2” indicate the left and right sides of the membrane located in the measuring cell filled with an electrolyte solution.

The membrane is characterized by the thickness *h*, the diffusion $D_{m+},\;D_{m-}$ and the equilibrium distribution coefficients $\gamma_{+},\;\gamma_{-}$ of cations and anions in the membrane matrix and the concentration $\left(-\bar{\rho }\right)$, $\bar{\rho }>0$, of fixed groups constant in its thickness (exchange capacity). Recall that $\gamma_{\pm }=\exp\left(\Phi_{\pm }\right)$ reflects the degree of interaction of ions with the membrane pore walls ($\Phi_{\pm }$: dimensionless potentials of the interaction of ions with the membrane pore walls in $k_{B}T$ units, $k_{B}$: Boltzmann constant). We also introduce the coefficient of the equilibrium distribution of a pair of ions in a membrane, $\gamma_{m}=\sqrt{\gamma_{+}\gamma_{-}}.$

Thermodynamic forces are set independently in the experiment. As dependent thermodynamic parameters measured in the experiment, we will use the flux densities of (1) solvent U, (2) mobile charges (electric current density) I, and (3) solute (density of the diffusion flux of the electrolyte) J (Figure 1). Then the phenomenological transport equations take the form:

(1) $$\left\{ \begin{array}{l} U=-\left(L_{11}\nabla p+L_{12}\nabla \varphi +L_{13}\nabla \mu \right),\\ I=-\left(L_{21}\nabla p+L_{22}\nabla \varphi +L_{23}\nabla \mu \right),\\ J=-\left(L_{31}\nabla p+L_{32}\nabla \varphi +L_{33}\nabla \mu \right). \end{array}\right.$$

Kinetic coefficients $L_{ij}$ were calculated in [32,33,34,35] using the cell model that considers the heterogeneous structure of the membrane. The cell model has been successfully verified on different types of ion-exchange membranes and electrolytes [30,31].

Here we will consider only the transfer of solvent (water) through a charged membrane under the combined action of gradients of electrical and chemical potentials and the absence of a pressure drop across the membrane:

(2) $$U=-L_{12}\nabla \varphi -L_{13}\nabla \mu$$

It follows from relation (2) that, depending on the directions of the gradients of the electrical and chemical potentials, the solvent transfer rate *U* can change sign since the current density, in this case, is equal to $I=-L_{22}\nabla \varphi -L_{23}\nabla \mu \;$ and, therefore $-L_{12}\nabla \varphi =\frac{L_{12}}{L_{22}}\left(I+L_{23}\nabla \mu \right)$. Substituting the last expression in relation (2), we obtain the following formula for the solvent transfer rate *U*:

(3) $$U=\frac{L_{12}}{L_{22}}I+\left(L_{23}\frac{L_{12}}{L_{22}}-L_{13}\right)\nabla \mu$$

We introduce permeabilities at a constant value of direct current:

(4) $$D_{I}=\frac{L_{12}}{L_{22}}$$

And at a constant gradient of chemical potential (concentration):

(5) $$D_{C}=\left(L_{23}\frac{L_{12}}{L_{22}}-L_{13}\right)\frac{RT}{C_{0}}=\left(L_{23}D_{I}-L_{13}\right)\frac{RT}{C_{0}}$$

Since $\mu =\mu_{0}+RT\ln\left(1+\frac{C}{C_{0}}\right)\approx \mu_{0}+RT\frac{C}{C_{0}}\;\;\Rightarrow \;\nabla \mu \;=\frac{RT}{C_{0}}\nabla C$, then

(6) $$U=D_{I}I+D_{C}\nabla C$$

Formula (6) allows calculating the solvent flux through the membrane when applying both direct current and a concentration drop. At the same time, for the electroosmotic and osmotic permeability of an ideal negatively charged (cation-exchange) membrane, we have the following formulae, respectively,

(7) $$D_{I}=\frac{3}{F_{0}{\bar{\rho }}_{0}}\frac{3-m_{0}\left(1-\frac{D_{m+}}{D_{+}}\right)}{\left(\frac{D_{m+}}{D_{+}}+\frac{\bar{\rho }}{{\bar{\rho }}_{0}}\right)\left(9\left(1-m_{0}\right)+2m_{0}^{2}\left(1+\frac{D_{-}}{D_{+}}\right)\right)+2m_{0}\left(1+\frac{D_{-}}{D_{+}}\right)\left(3-m_{0}\left(1+\frac{\bar{\rho }}{{\bar{\rho }}_{0}}\right)\right)\frac{C_{0}}{\bar{\rho }}}$$

(8) $$D_{C}=\frac{3D_{+}}{2{\bar{\rho }}_{0}}\left(\begin{array}{l} \frac{1}{\left(3-m_{0}\right)}\left(2m_{0}\left(1-\frac{D_{-}}{D_{+}}\right)+\frac{9\left(1-m_{0}\right)\left(\frac{D_{m+}}{D_{+}}+\frac{\bar{\rho }}{{\bar{\rho }}_{0}}\right)}{m_{0}\left(\frac{D_{m+}}{D_{+}}+\frac{\bar{\rho }}{{\bar{\rho }}_{0}}\right)+\left(3-m_{0}\left(1+\frac{\bar{\rho }}{{\bar{\rho }}_{0}}\right)\right)\frac{C_{0}}{\bar{\rho }}}\right)\cdot \\ \frac{3-m_{0}\left(1-\frac{D_{m+}}{D_{+}}\right)}{\left(\frac{D_{m+}}{D_{+}}+\frac{\bar{\rho }}{{\bar{\rho }}_{0}}\right)\left(9\left(1-m_{0}\right)+2m_{0}^{2}\left(1+\frac{D_{-}}{D_{+}}\right)\right)+2m_{0}\left(1+\frac{D_{-}}{D_{+}}\right)\left(3-m_{0}\left(1+\frac{\bar{\rho }}{{\bar{\rho }}_{0}}\right)\right)\frac{C_{0}}{\bar{\rho }}}-\\ \frac{1}{m_{0}\left(\frac{D_{m+}}{D_{+}}+\frac{\bar{\rho }}{{\bar{\rho }}_{0}}\right)+\left(3-m_{0}\left(1+\frac{\bar{\rho }}{{\bar{\rho }}_{0}}\right)\right)\frac{C_{0}}{\bar{\rho }}} \end{array}\right)$$

where *F*_0_ is the Faraday constant, $D_{+},\;\;D_{-}$ are the diffusion coefficients of cations and anions in the electrolyte solution at infinite dilution, *m*_0_ is the macroscopic porosity of the membrane, ${\bar{\rho }}_{0}=\frac{\mu^{o}D_{+}}{k_{D}RT}\;$ is the characteristic exchange capacity of the membrane system [32,33,34,35], *k*_D_ is the specific hydrodynamic permeability of the ionite grain (gel), *µ*^o^ is the viscosity of the solution.

In the general case of non-ideal membranes, formulae that are analogous to (7) and (8) are significantly complicated and can be obtained using expressions (4) and (5) and the kinetic coefficients $L_{12},\;\;L_{22},L_{23},\;L_{13}$ calculated in [32,34,35].

Formula (6), when divided by a molar volume of water, $\vartheta_{H_{2}O}=0.018\;\;{\mathrm{dm}}^{3}/\mathrm{mol}\;$ gives a molar flux of solvent (water):

(9) $$j_{w}=t_{w}^{e.o.}\left(\frac{I}{F_{0}}\right)+t_{w}^{\mathrm{os}.}\left(D_{+}\nabla C\right)$$

where the electroosmotic number of water transport is introduced,

(10) $$t_{w}^{e.o.}=D_{I}\frac{F_{0}}{\vartheta_{H_{2}O}}$$

for an ideal membrane equal to

(11) $$t_{w}^{e.o.}=\frac{3}{{\bar{\rho }}_{0}\vartheta_{H_{2}O}}\frac{3-m_{0}\left(1-\frac{D_{m+}}{D_{+}}\right)}{A\left(9\left(1-m_{0}\right)+2m_{0}^{2}D\right)+2m_{0}DB}$$

and osmotic number of water transport,

(12) $$t_{w}^{\mathrm{os}.}=\frac{D_{C}}{D_{+}\vartheta_{H_{2}O}}$$

for the same membrane equal to

(13) $$t_{w}^{\mathrm{os}.}=\frac{3}{2{\bar{\rho }}_{0}\vartheta_{H_{2}O}}\left(\frac{2m_{0}\left(2-D\right)+\frac{9\left(1-m_{0}\right)A}{m_{0}A+B}}{3-m_{0}}\cdot \frac{3-m_{0}\left(1-\frac{D_{m+}}{D_{+}}\right)}{A\left(9\left(1-m_{0}\right)+2m_{0}^{2}D\right)+2m_{0}DB}-\frac{1}{m_{0}A+B}\right)$$

Here, for the sake of brevity, new dimensionless parameters are introduced:

(14) $$A=\frac{D_{m+}}{D_{+}}+\frac{\bar{\rho }}{{\bar{\rho }}_{0}}>0,\;\;B=\left(3-m_{0}\left(1+\frac{\bar{\rho }}{{\bar{\rho }}_{0}}\right)\right)\frac{C_{0}}{\bar{\rho }}>0,\;\;D=1+\frac{D_{-}}{D_{+}}>1$$

From expressions (11) and (13), it is possible to obtain a relationship between water transport numbers having different natures:

(15) $$t_{w}^{\mathrm{os}.}=\left(\frac{t_{w}^{e.o.}}{\left(3-m_{0}\right)}\left(m_{0}\left(2-D\right)+\frac{\frac{9}{2}\left(1-m_{0}\right)A}{m_{0}A+B}\right)-\frac{3}{2{\bar{\rho }}_{0}\vartheta_{H_{2}O}}\frac{1}{m_{0}A+B}\right)$$

## 3. Discussion

**Figure 2:**
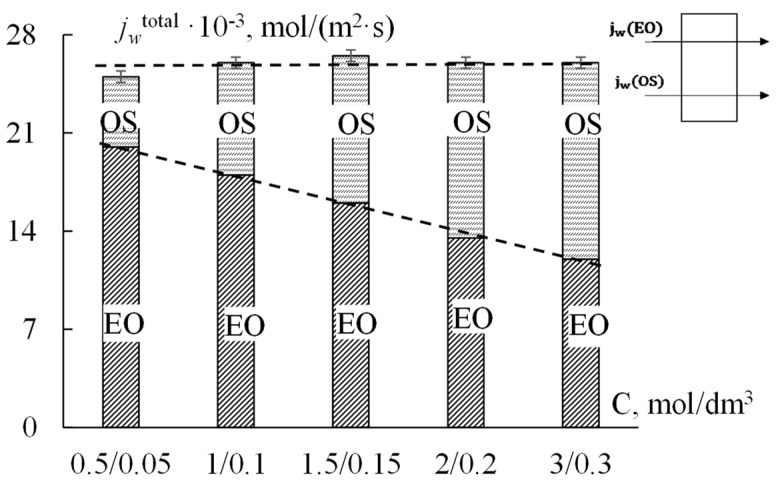
The total water flux through the MF−4SC membrane in NaCl solutions with the concentration ratio *C*_10_/*C*_20_ = 10: OS and EO are the contributions of osmotic and electroosmotic mechanisms of water transport, respectively.

The obtained experimental data for the total water flux and the contributions of osmotic and electroosmotic mechanisms of water transport through the extrusion MF−4SC p.29 membrane are presented in Figure 2 in the form of a histogram. As can be seen from Figure 2, the total water flux through the MF−4SC cation-exchange membrane in NaCl solutions with a constant concentration ratio on both sides of the membrane equal to 10 does not change with an increase in the concentration gradient (from 0.5/0.05 to 3/0.3). However, it was found that the contributions of osmotic and electroosmotic fluxes to total water transport have changed. An increase in the concentration difference from 0.45 to 2.7 M leads to an increase in the osmotic flux of water by 60%. At the same time, the contribution of the electroosmotic flux to the total water flux decreases. This is consistent with experimental data obtained by direct measurement of water transport numbers [36], in which, with increasing concentration, the number of water transport with the Na^+^ ion decreases by almost 40%. This is due to a decrease in the amount of water in the hydrate shell of the sodium ion with an increase in the concentration of the external electrolyte solution.

Thus, for the MF−4SC p.29 membrane, the contribution of the electroosmotic component to the total water flux was calculated, which decreases from 70% to 45% with an increase in the difference of solution concentrations on both sides of the membrane from 0.45 to 2.7 M. Under these conditions, the value of the osmotic water flux increases by 40%, and the electroosmotic flux decreases by 30–40%.

**Figure 3:**
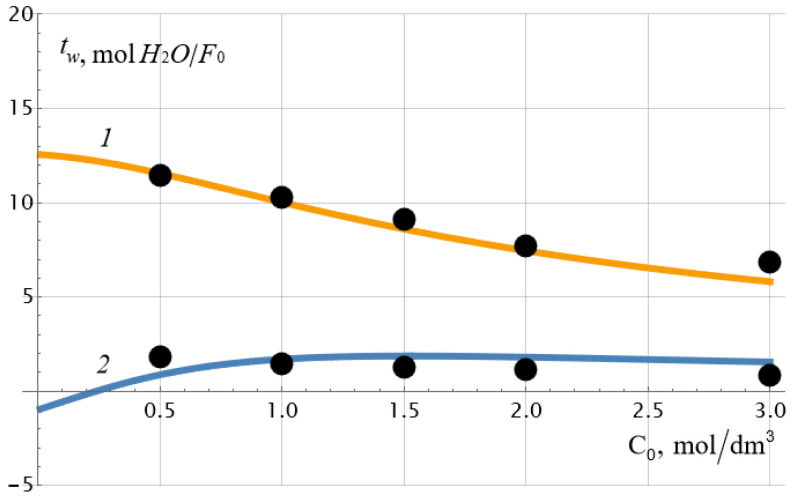
Experimental (symbols) and theoretical values of the electroosmotic (curve *1*) and osmotic (curve *2*) numbers of water transport through the MF−4SC membrane depending on the equilibrium concentration of NaCl aqueous solution in the case of co-directional electroosmotic and osmotic water fluxes.

The experimental data presented in Figure 2 were calculated using the cell membrane model [30,31,32,33,34,35] and independently obtained data on the ion-exchange capacity of the MF−4SC p.29 membrane equal to $\bar{\rho }$ = 1.105 mol/dm^3^ and its macroscopic porosity *m*_0_ = 7.7%. The remaining physicochemical parameters of the cell model were found using a special procedure for the one-time minimization of the deviation of the experimental values of the electroosmotic and osmotic transport numbers from their theoretical values. To do this, we used exact formulae for the specified transport numbers, which are not given here due to their bulkiness. The numerical procedure is similar to the one described in our paper [30]. Approximate expressions of water transport numbers (11) and (13) can be used at the stage of preliminary estimation of their values. Table 1 shows the values of the given and calculated parameters of the membrane system, and Figure 3 shows the results of comparing experimental and theoretical dependences of the water transport numbers on the electrolyte concentration (NaCl). The arithmetic mean of the concentrations on the left (*C*_10_) and on the right (*C*_20_) sides of the membrane was chosen as the equilibrium concentration *C*_0_.

**Figure 4:**
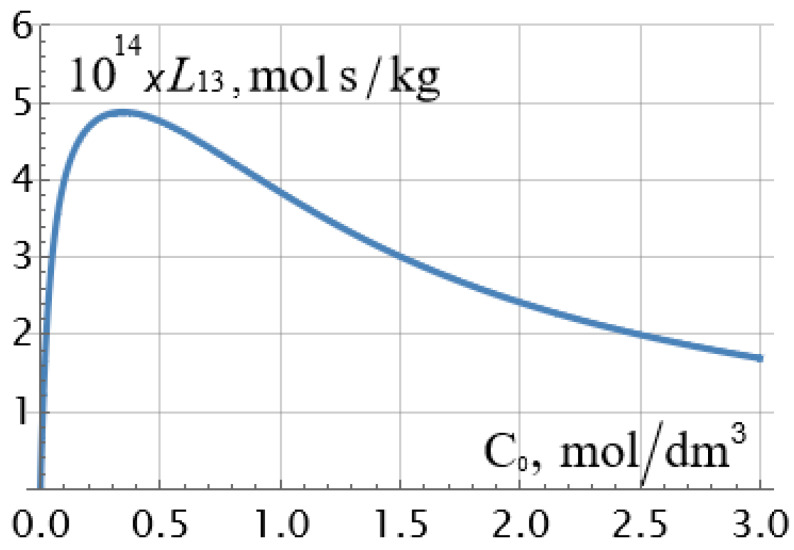
Theoretical values of the true osmotic permeability *L*_13_ of the MF−4SC membrane depend on the equilibrium concentration of an aqueous solution of NaCl.

As can be seen from Table 1, ${\bar{\rho }}_{0}$ exceeds the value of the exchange capacity $\bar{\rho }$ of the membrane by about 5 times, and the diffusion coefficient of the sodium cation in the membrane is more than an order of magnitude higher than the diffusion coefficient of the chlorine ion so that the diffusion coefficient of the sodium chloride molecule in the membrane is equal to $D_{m}=\frac{2D_{m+}D_{m-}}{D_{m+}+D_{m-}}=147\;{\mu m}^{2}/s$. This value is 11 times lower than the diffusion coefficient of the NaCl molecule in a dilute solution: $D=\frac{2D_{+}D_{-}}{D_{+}+D_{-}}=1622\;{\mu m}^{2}/s$, which is quite consistent with the physical concepts of a decrease in the diffusion coefficient of the electrolyte molecule by at least an order of magnitude when introduced into a porous medium. It may seem that the value of osmotic permeability cannot be negative, as is in the case for the osmotic theoretical curve 2 in Figure 3. And this is indeed the case if we are thinking about the true osmotic permeability coefficient *L*_13_, the dependence of which on the concentration is plotted in Figure 4 for a set of parameters from Table 1. However, it should be recalled that when calculating *L*_13_, there should be no pressure and electric potential differences on the membrane, and the potential chemical difference should remain constant. At the same time, it follows from formulae (5) and (13) that under constant current density and constant concentration gradient, osmotic permeability and osmotic transport number of water can have negative values at low electrolyte concentrations. The second reason for this unexpected effect may be the insufficient accuracy of the applied theory at very low concentrations when the thickness of the electrical double layers cannot be neglected. Note that the monotonous drop in the electroosmotic number of water transport with an increase in the equilibrium concentration of the electrolyte, recorded in Figure 3 (curve *1*), was also observed for the heterogeneous cation-exchange membrane MK-40, studied in the work already mentioned above [9]. As can be seen in Figure 3, there is a good correspondence between theory and experiment.

**Figure 5:**
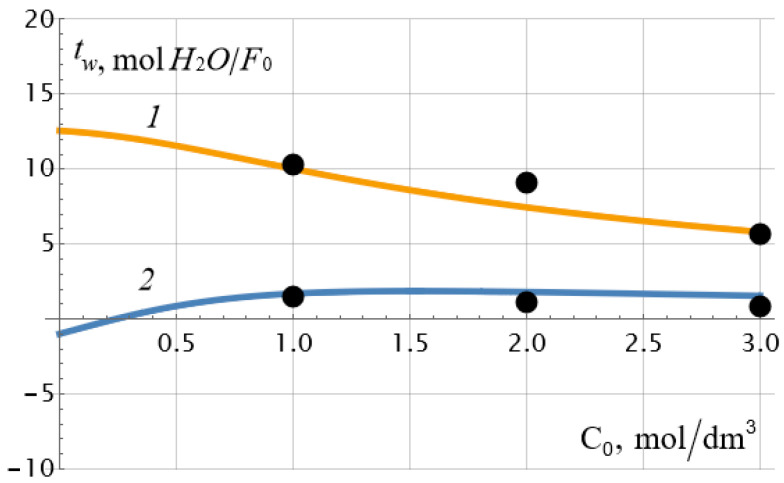
Experimental (symbols) and theoretical values of the electroosmotic (curve *1*) and osmotic (curve *2*) numbers of water transport through the MF−4SC p.29 membrane depending on the equilibrium concentration of an aqueous solution of NaCl in the case of counter-directional electroosmotic and osmotic water fluxes.

The same parameter values (Table 1) were used to calculate theoretical values of transport numbers of water in the membrane system for counter-directional electroosmotic and osmotic fluxes (Figure 5). Experimental values of counter-directional fluxes were obtained by reversing the electric field through the cell. In this case, the electric current density in formulae (6) and (9) should be considered negative, and the concentration gradient positive.

As can be seen from Figure 5, the experimental points are located in the vicinity of the theoretical curves, which confirms the complete adequacy of the theory. In the case under consideration, there are no fitting parameters at all for the theoretical values of both osmotic and electroosmotic water transport numbers.

It should be noted that the developed water transfer model is applicable to any charged membranes, including those used in electrobarofiltration. In particular, our cell model will allow calculating water transfer due to the simultaneous action of pressure and electrical and chemical potential gradients when using promising electrospun nanofibrous membranes utilized for water and wastewater treatment [37].

## 4. Materials and Methods

The object of research was extrusion perfluorinated sulfonated cation-exchange MF−4SC p.29 membrane (JSC Plastpolymer, St. Petersburg, Russia). The thickness of the membrane was equal to *h* = 220 microns.

Deionized water and NaCl (LLC Trading House “KhimMed”, Moscow, Russia, extra-pure grade) were used in this experiment to obtain the salt solutions of the needed concentration.

**Figure 6:**
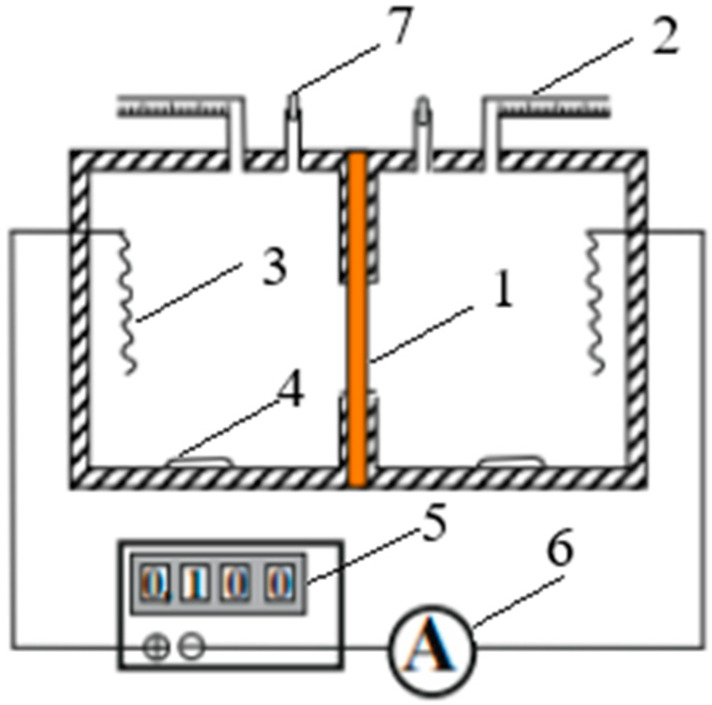
An electrical circuit and a cell for determining the water fluxes through the membrane: 1—the membrane under study, 2—measuring capillaries, 3—silver-silver chloride electrodes, 4—magnetic stirrers, 5—a DC source, 6—an ammeter, 7—funnels for filling the cell with a working solution.

The measurement of the total water flux is made based on the change in solution volume when passing a certain current through the investigated membrane (1). The cell consisting of two compartments equipped with measuring graduated capillaries (2) is shown in Figure 6. The volume of the compartments is 200 cm^3^. The Ag/AgCl polarizing electrodes (3) were incorporated into the cell chambers. The cell hermetic was achieved by means of thin rubber spacers between the cell chambers and was tested after each filling with electrolyte solution through funnels (7), the cell being without current flow for 30 min. The ratio of electrolyte concentrations on different sides of the membrane was 10. To reduce the possibility of concentration polarization, magnetic stirrers (4) were used in the cell chambers. Special attention during the experiments was paid to the state of reversibility of the polarizing electrodes (3). The investigated membrane was fixed between the cell chambers, and the system was checked for pressurizing; after that, a direct current was passed through the membrane from a DC source (5), and the changes in the liquid volume in the capillary tubes (2) were registered as a function of time. The meniscus movements were followed in both capillaries and gave results for the rate of the water flux, which agreed within a 1% error. Measurements were repeated at each concentration several times, and the current was inverted after each run.

So, the total water flux $j_{w}^{\mathrm{total}}$ across a membrane in NaCl solutions was determined by the volumetric method in the two-chamber cell (Figure 6) with the simultaneous action of electric and concentration fields.

The obtained values of the change in the volume of solution *V* in the capillaries of the cell at given current density *I* during the time *τ* were used to calculate the total $j_{w}^{\mathrm{total}}$ water flux according to the formula:

(16) $$j_{w}=\frac{V}{S\tau \vartheta_{H_{2}O}}$$

where *S* is the working area of the membrane (in experiments, it was assumed to be equal to 1.778 cm^2^). The current strength (30 mA) was kept constant, which was equivalent to the current density *I* = 168.7 A/m^2^.

Osmotic flux $j_{w}^{\mathrm{os}.}$ was determined in the same cell (Figure 6) under the conditions of the concentration gradient alone: the ratio of electrolyte concentrations *C*_10_/*C*_20_ on different sides of the membrane was equal to 10. The osmotic flux was calculated from Equation (16) also using the obtained experimental data of the change in the volume of solution *V* inside the capillaries.

**Figure 7:**
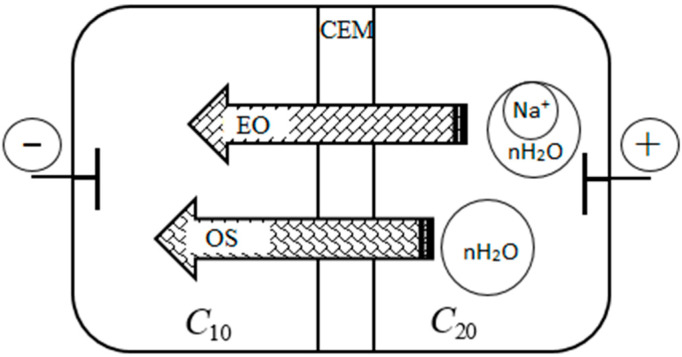
Schematic representation of co-directional electroosmotic (EO) and osmotic (OS) water fluxes through a cation-exchange membrane (CEM) in NaCl solutions.

Real electromembrane processes of concentrating electrolytes are carried out both in concentration and in electric fields. As a result, various fluxes appear in the electromembrane cell: diffusion, electroosmotic, and osmotic. Figure 7 schematically shows the transfer of water through the membrane by electroosmotic $j_{w}^{e.o.}$ and osmotic $j_{w}^{\mathrm{os}.}$ mechanisms under conditions of electrodialysis concentrating. The diagram shows that the electroosmotic flux of water is directed in accordance with the external electric field, and the osmotic flux is in accordance with the concentration field. At the same time, under conditions of electrodialysis concentrating, these fluxes are co-directed into the concentration chamber of the electrodialysis cell. Thus, the total water flux $j_{w}^{\mathrm{total}}$ consists of two fluxes:

(17) $$j_{w}^{\mathrm{total}}=j_{w}^{e.o.}+j_{w}^{\mathrm{os}.}$$

The electroosmotic flux $j_{w}^{e.o.}$ was calculated from the obtained experimental data of the total and osmotic fluxes using Equation (17).

## 5. Conclusions

Based on the cell model of a charged membrane, analytical formulae for calculating the osmotic and electroosmotic permeability of the membrane in aqueous solutions of 1:1 electrolyte at constant electric current density and constant concentration gradient are suggested, which are exact for a real membrane and approximate for an ideally selective membrane. The exact formulae obtained have been successfully verified on our own experimental data for the extrusion cation exchange membrane MF−4SC p.29 in NaCl solution up to concentrations of 3 M. The cases of co- and counter-directed osmotic and electroosmotic water fluxes are considered. A good correspondence between theoretical and experimental results was obtained, which made it possible to determine the physicochemical parameters of the electromembrane system (diffusion coefficients of individual ions and the coefficient of equilibrium distribution of electrolyte molecules inside the membrane matrix, the characteristic exchange capacity of the cell model). The proposed model can be easily used to calculate osmotic and electroosmotic water transfer through an arbitrarily charged membrane (i.e., ion exchange, reverse osmosis, nano- and ultrafiltration membranes) with the combined superposition of electric and concentration fields. The results obtained here make it possible to completely characterize existing and promising types of ion exchange and other charged membranes based on the developed cell model of a charged membrane.

## Figures and Tables

**Table 1:** Parameters of the MF−4SC extrusion p.29 membrane in NaCl solution.

*m*_0_, %	$\bar{\rho }$, mol/dm^3^	*h*, µm	*γ*_m_	${\bar{\rho }}_{0}$, mol/dm^3^	*D*_m+_, µm^2^/s	*D*_m-_, µm^2^/s
7.7	1.105	220	0.95	5.23	915	80

## Data Availability

Not applicable.
